# BUTYRATE-PRODUCING BACTERIA INCREASE GUT MICROBIOTA DIVERSITY TO DELAY INTESTINAL INFLAMMAGING

**DOI:** 10.1093/geroni/igad104.2990

**Published:** 2023-12-21

**Authors:** Xiaoang Li, Yiming Zhang, Liping Duan

**Affiliations:** Peking University Third Hospital, Beijing, Beijing, China (People’s Republic); Peking University Third Hospital, Beijing, Beijing, China (People’s Republic); Peking University Third Hospital, Beijing, Beijing, China (People’s Republic)

## Abstract

Intestinal flora dysbiosis is a key link in intestinal inflammaging, and the diversity of gut microbiota plays an important role in maintaining the entire intestinal microecology. Our previous study found that butyrate-producing bacteria reversed aging-induced intestinal inflammation and promoted the expression of intestinal anti-inflammatory factors in 24-month-old mice, but the mechanism was unclear. We found that butyrate-producing bacteria promote the expression of short-chain fatty acid (SCFA) receptors in the intestine. Monocarboxylate transporter 1 (MCT-1) has anti-inflammatory effects on intestinal inflammation. We speculate that butyrate-producing bacteria upregulate MCT-1 expression through the activation of SCFA receptors, thereby promoting gut stability and bacterial diversity. In further experiments, we established inflammation models using cell lines, colon organoids, mouse colonic epithelial cells, and aged mice (24 months old) given different concentrations of LPS and DSS, and then added butyrate-producing bacteria to verify that MCT-1 is involved in the resistance of butyrate-producing bacteria to intestinal inflammation. We found that bacteria up-regulated the expression of MCT-1 by activating SCFA receptors. From the results of 16s rRNA sequencing, it can be seen that the intestinal stability and bacterial diversity of aged mice were improved after the administration of butyrate-producing bacteria. The research results will provide an experimental basis for treating intestinal inflammaging by restoring intestinal stability and diversity of gut microbiota and will help to explore the clinical value of butyrate-producing bacteria, which has important translational significance.

